# The role of CircRNA/miRNA/mRNA axis in breast cancer drug resistance

**DOI:** 10.3389/fonc.2022.966083

**Published:** 2022-09-05

**Authors:** Mohammad H. Ghazimoradi, Sadegh Babashah

**Affiliations:** Department of Molecular Genetics, Faculty of Biological Science, Tarbiat Modares University, Tehran, Iran

**Keywords:** breast cancer, circRNA, miRNA, drug resistance, oncogenes, tumor suppressors

## Abstract

Multidrug resistance is one of the major obstacles in the treatment of cancers. This undesirable feature increases the mortality rate of cancers, including breast cancer. Circular RNA (CircRNA)/microRNA (miRNA)/messenger RNA (mRNA) is one of the important axes with major roles in the promotion and resistance of breast cancer. This heterogeneous pathway includes mRNA of oncogenes and tumor suppressors, which are controlled by miRNAs and CircRNAs. Unfortunately, this network could be easily deregulated, resulting in drug resistance and tumor development. Therefore, understanding these dysregulations may thus help to identify effective therapeutic targets. On this basis, we try to review the latest findings in the field, which could help us to better comprehend this significant axis in breast cancer.

## 1. Introduction

**Figure 1:**
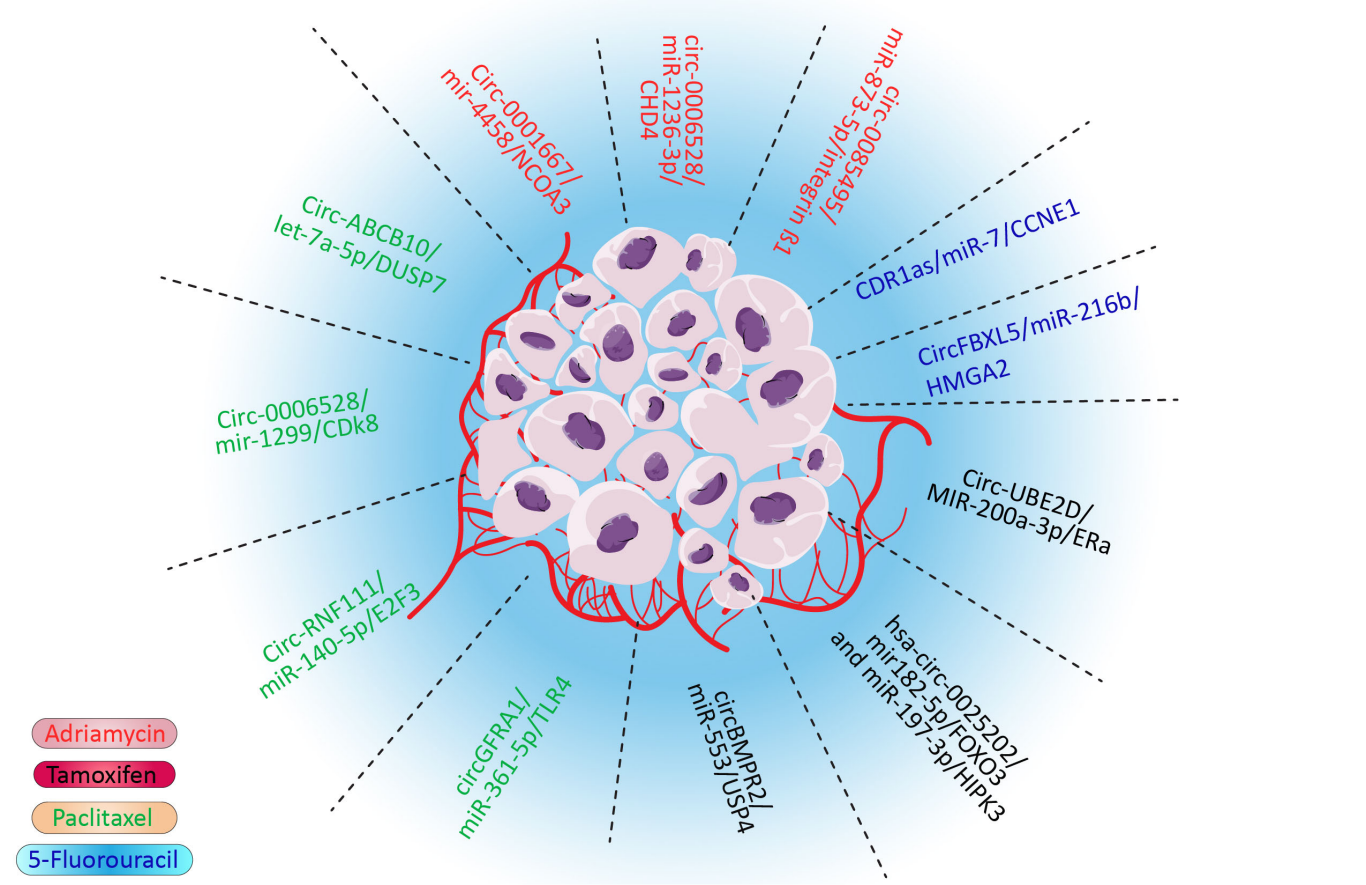
Summary of significant drug resistance in the CircRNA/miRNA/mRNA axis in breast cancer.

Drug resistance is one of the major hindrances in treating cancers, including breast cancer, which leads to treatment failure and a higher mortality rate (1, 2). Various drugs, such as doxorubicin (3), tamoxifen (4), paclitaxel (5), and even targeted therapies, could develop drug resistance in cancers (6). Although multidrug resistance (MDR) is a major impediment in the treatment of breast cancer, the molecular pathways remain to be studied. Several mechanisms of MDR have been discovered. These mechanisms give cancer cells the ability to tolerate cancer cells. For example, cancer cells could flux anticancer drugs *via* deregulated molecular pumps or hinder the access of drugs to cancer cells (7). Furthermore, various survival pathways could be influenced in cancer cells, which would give them survival advantages. This pathway also confers the drug mechanisms by deregulating molecular pathways that the drug affects, such as EMT, apoptosis, and DNA repair pathways (8). It was also proposed that CSCs could overcome anticancer drugs due to their characteristics, such as lower metabolisms, self-renewal, and differentiation ability (9). Although these proposed mechanisms could explain MDR, various aspects of MDR remain to be discovered. Noncoding RNA is one of the most important regulatory mechanisms in cells. Most of the transcriptome is made by these RNAs. These RNAs could regulate multiple targets, which demonstrates their importance (10, 11). Circular RNA is one of the most important noncoding RNAs. Circular RNA is highly stable and crosses paths with various significant pathways such as MAPK/ERK and PTEN/PIK3/AKT pathways (12). These looped RNAs played their roles *via* various mechanisms, including binding to proteins, sponging microRNAs (miRNAs), and interfering with the splicing of other RNAs. The sponging of miRNA to microRNA response elements of circular RNA is one of the most influential and deregulated mechanisms in cancer (12). In this pathway, circular RNA (CircRNA) sponges the miRNA and inhibits their role as silencers of oncogene messenger RNA (mRNA) or interferes with tumor suppressors by deregulation of their natural pathways and in drug resistance–related gene (13). Notably, the silencing role of microRNAs is not limited to degradation of mRNA as they have various roles, including modulation of gene expression and transactional repression (14). As miRNAs have thousands of targets *via* various mechanisms and circular RNA could sponge to tens of these regulatory RNAs, even the slightest deregulation in the CircRNA/miRNA/mRNA pathway could be catastrophic (15). It has been shown that CircRNA/miRNA/mRNA could interfere with doxorubicin (3), tamoxifen (4), paclitaxel, 5-fluorouracil, and many more drugs (**Figure 1**). Although this impactful pathway has been studied vigorously, its role in drug resistance, especially in breast cancer, needs to be reviewed. By comprehending this pathway in drug resistance, potential therapeutic targets will be obtained to overcome MDR.

## 2. Circular RNAs

Most of the human transcriptome is made up of noncoding RNAs. This category has a regulatory role in humans. One of the most significant subgroups of noncoding RNAs is circular RNA. As in their name, the circular formation of these RNAs protects them from RNase R or RNA exonucleases (16). This feature results in more stable RNA and, consequently, more impactful regulatory elements. Circular RNA is classified into three types based on its biogenesis and structure: circRNA from exons (ecircRNA), circRNA with 3′,5′- or 2′,5′-phosphodiester bond (ciRNA), and circRNA from exon–intron junctions (EIciRNA) (17). These regulatory factors have various roles in biological systems, from metabolism to diseases (18–20). CircRNA mechanism of action is distinct. They could act as sponges for other regulatory RNAs. These elements could regulate splicing or attach to proteins and guide them (21). Even though these RNAs are noncoding, some peptides and proteins have been seen on special occasions with significant roles (22). The role of this regulatory RNA in cancer is becoming clearer. Many circular RNAs have crossed paths with signaling, invasion, and metastasis pathway; many researchers devote their time to this subject (23). Drug resistance is a significant phenomenon in clinical outcomes. Although the role of circular RNA in the progression of cancer has been studied, its role in drug resistance is under investigation.

## 3. Circular RNA/miRNA/mRNA pathway

**Figure 2:**
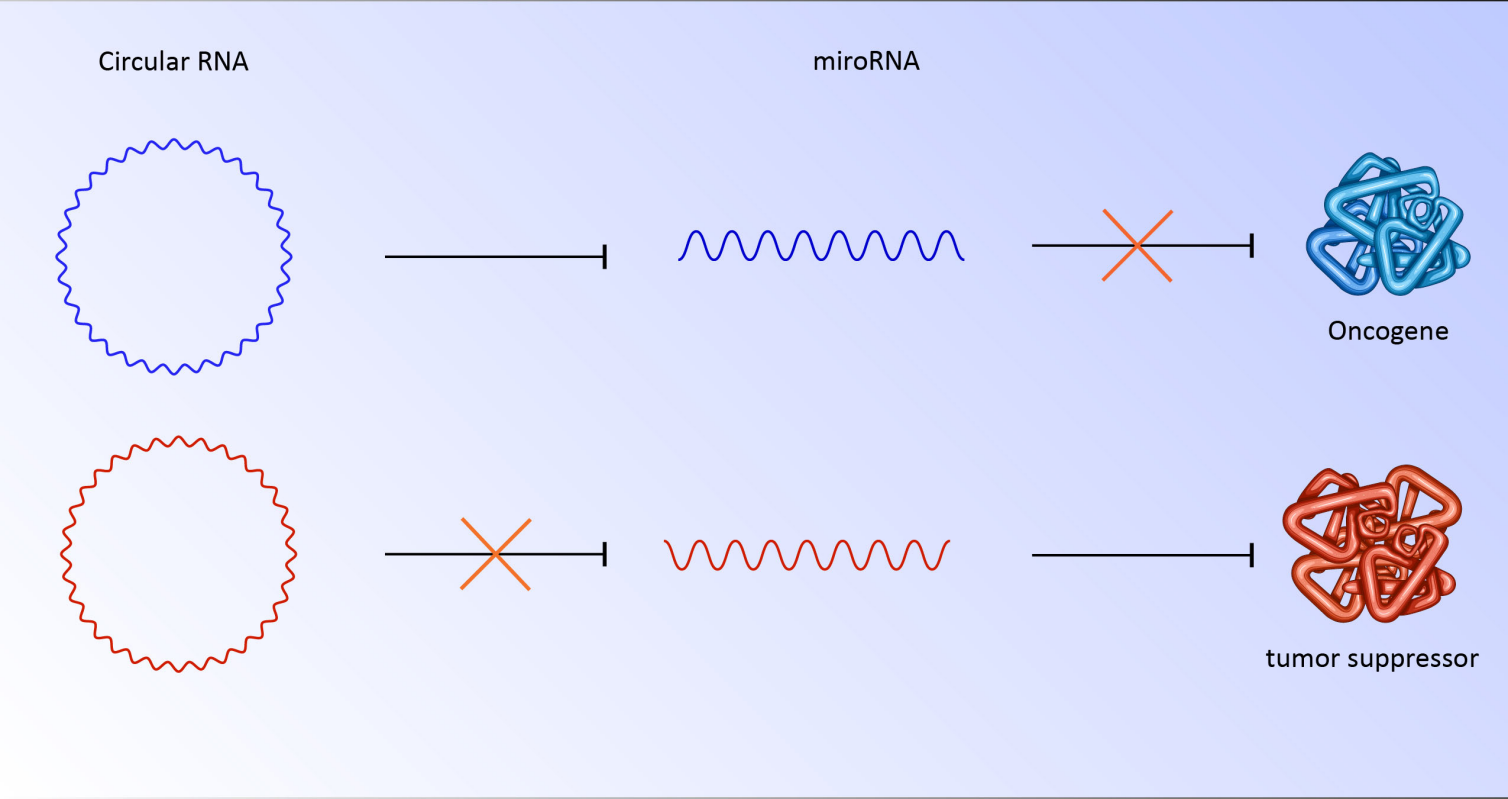
CircRNA/miRNA/mRNA potential pathway for progression of breast cancer.

Circular RNA has various roles in the regulation of genes and proteins. Although these RNAs perform their role by various mechanisms, a substantial number of circular RNAs are rich in miRNA response elements (MREs) (24, 25). miRNAs are 22 nucleotide regulatory RNAs. By targeting the 3′-UTR, this element may be able to regulate posttranscriptional gene expression. This interaction could suppress the translation or stability of mRNA. The binding of miRNAs to MREs of circular RNA hinders them from their regulatory roles, which results in the translation of their downstream targets (26). For instance, CDR1as has more than 70 binding sites for miR-7. As a result, deregulation of circular RNA could strongly affect miRNA and its downstream targets (27). In cancer cells, abnormally overexpressed or downregulated circular RNA has been seen repeatedly. The overexpression of circs could sponge miRNA responsible for targeting oncogenes (27, 28). It is also possible that downregulation of circ unleashes miRNA which targets tumor suppressors (**Figure 2**). Notably, these RNAs could be transported to other cells by exosomes, which have clinical implications for metastasis and niching of cancer cells (28, 29). Various examples of deregulated circular RNA have been reported, including circRNA ZNF609 (30), circ-SRY (31), mm9_circ_012559 (32), and circDOCK1 (33) with potential roles in the promotion of invasion, metastasis, and proliferation of cancer cells.

## 4. The role of circular RNA in drug resistance breast cancer

### 4.1. Adriamycin

Doxorubicin (Adriamycin), an anthracycline chemotherapy agent, is the most effective prescription for breast cancer treatment. Although multidrug resistance in breast cancer often leads to chemotherapy failure, doxorubicin is not an exception. Doxorubicin resistance results in poor prognosis and survival (34, 35). As the doxorubicin mechanism of action is related to topoisomerase II and linking double-strand DNA, various molecular pathways in the apoptosis pathway could lead to drug resistance status (36). Surprisingly, all of the pathways that lead to the doxorubicin resistance phenotype are linked to the acetylation of histones such as CDH4 (37) and nuclear receptor coactivator 3 (NCOA3) (38). CHD4 is the most important molecule in the nucleosome remodeling and deacetylase complex, and NCOA3 is an activator protein with acetyltransferase intrinsic activity, which both are affected by Adriamycin resistance. It has been shown that integrin β1 overexpression leads to p21WAF1/Cip1 transcription *via* p300-mediated histone acetylation (39). Histone H1 hyperacetylation has also been seen in resistant colon cancer cells but not in sensitive cells. This evidence could implicate that acetylation of chromatin could play an important role in doxorubicin resistance.

#### 4.1.1. Circ_0085495/miR-873-5p/integrin β1

Circ_0085495, located in the cytoplasm, is upregulated in doxorubicin resistance breast cancer cells. This stable circular RNA by sponging miR-873-5p enhances Doxorubicin resistance, proliferation, and metastasis of ADM-resistant breast cancer cells. Furthermore, miR-873-5p negatively controls the expression of integrin β1, an important gene in the regulation of metastasis, proliferation, and drug resistance *via* various molecular pathways. The knockdown of this circular RNA attenuates the proliferation and invasion of BC cells *in vitro*. The negative effects of Circ_0085495 knockdown could be overcome by miR-873-5p overexpression or integrin β1 inhibition. Moreover, repressing Circ_0085495 could inhibit tumor growth and increase tumor cell sensitivity to doxorubicin *in vivo* (39).

#### 4.1.2. Circ_0006528/miR-1236-3p/CHD4

miR-1299 is a downstream target of circ_0006528 in BC. The expression of miR-1299 is downregulated in doxorubicin-resistant breast cancer cell lines and tissues, while a sharp upregulation has been seen for circ_0006528 and CHD4 in contrast with sensitive cancer cells. circ_0006528 has oncogenic effects *via* sponging of miR-1236-3p and increasing CHD4, a downstream target of miR-1299 and an activator of the RhoA/ROCK pathway. This circular RNA also has an important role in tamoxifen resistance cells in BC. Furthermore, knockdown of circ_0006528 could decrease the IC50 of paclitaxel-resistant cells and attenuate the proliferation, migration, invasion, and autophagy of these cells. Moreover, the knockdown of circ_0006528 is solely responsible for the induction of apoptosis in BC cells (40).

#### 4.1.3. Circ_0001667/miR-4458/NCOA3

The NCOA3 is a transcriptional coactivator protein with acetyltransferase ability. This protein has an undeniable role in the endocrine therapy resistance to HER2/neu overexpression, activating mutations in PIK3CA (PI3K), and activating mutations in the proto-oncogene tyrosine-protein kinase Src (41). NCOA3 is the target of miR-4458, as proved by luciferase assay. miR-4458 has been sponged by Circ_0001667. It has been shown that Circ_0001667 and NCOA3 upregulate in tissues and cell lines, especially in resistant ones, while miR-4458 shows a reduction in expression. Cui et al. showed that the overexpression of miR-4458 or knockdown of Circ_0001667 and NCOA3 could increase the sensitivity of BC cells to doxorubicin. CircRNA_104889 is another circular RNA sponging miR-4458 and promoting lung adenocarcinoma cells (42).

### 4.2. Tamoxifen

Tamoxifen is a selective estrogen receptor modulator and the most widely used endocrine therapy for patients with hormone receptor (HR) positive breast cancer of the luminal subtype (43). Despite the significant efficiency, 40% of patients eventually resist this drug, leading to a decrease in drug effectiveness and patient survival (44).

#### 4.2.1. Circ_UBE2D2/MiR-200a-3p/ERα

Circ_UBE2D2, an exosomal circular RNA that can sponge different microRNAs, is overexpressed in resistant breast cancer tissues and cell lines. The knockdown of Circ_UBE2D2 could increase the susceptibility of cancer cells to tamoxifen. Invasion and migration of BC cells have also been decreased upon Circ_UBE2D2 knockdown *in vitro*. Introducing exosomes containing Circ_UBE2D2 enhances tamoxifen resistance, migration, and invasion of cells and could increase the size of tumors in mouse models. Furthermore, this RNA could negatively control E-CAD and ERα and, in contrast, enhance the expression of VIMENTIN. Of note, the introduction of miR-200a-3p, which is downregulated in resistance BC, could inhibit the expression of Circ_UBE2D2. Overexpression of miR-200a-3p attenuated exosomal effects of Circ_UBE2D2 on the increase of vimentin and the decrease of E-cad and ERα expressions. As it appears, Circ_UBE2D by binding to miR-200a could change the expression of key genes in EMT and increase the resistance status of cancer cells by controlling ERα (45).

#### 4.2.2. hsa_circ_0025202/mir-182-5p/FOXO3 and miR-197-3p/HIPK3

Hsa_circ_0025202 is a circular RNA that acts as a sponge for miR-182-5p and miR-197-3p, resulting in overexpression of Forkhead box class O 3 (FOXO3) and Homeodomain Interacting Protein Kinase 3 (HIPK3). FOXO3, known as a tumor suppressor, acts *via* decreasing cell proliferation and metastasis and increasing apoptosis in various cancers, including breast cancer (45); however, the role of HIPK3 has received less attention. It has been shown that hsa_circ_0025202 has low expression in tumoral tissue in comparison to normal tissues. hsa_circ_0025202 expression also shows a divergence relationship with lymphatic metastasis and histological grade. Overexpression of hsa_circ_0025202 Reversed the Progressive Phenotype and TAM Resistance *in vitro* and *in vivo*, while miR-182 and miR-197 mimic could reverse this process. Indeed, knockdown of FOXO3 and HIPK3 could mimic the phenotype of low expressed hsa_circ_0025202 BC cells and induce resistance status in MCF7 and T47D cells. Notably, CircFOXO3 *via* sponging of miR-138-5p and miR-432-5p could increase proliferation and invasion of GBM cells *in vitro* and *in vivovivo*, which shows the high value of FOXO3 gene and their subordinates in cancers (46, 47).

#### 4.2.3. CircBMPR2/miR-553/USP4

Ubiquitin-specific protease 4 (USP4) is a deubiquitinating enzyme that has important roles in signaling pathways including NF-κB, TGF-β, Wnt/β-catenin, and p53 (48). It has been shown that circBMPR2 *via* sponging of mir-553 could regulate USP4. CircBMPR2 also hinders the motility of breast cancer cells. Its expression in cancerous tissues is lower than in normal tissues. Loss and gain experiments show the role of circBMPR2 in the promotion of tamoxifen resistance of breast cancer cells *via* inhibiting apoptosis, and naturally overexpression of miR-553 could abort tumor suppressor (48).

### 4.3. Paclitaxel

Paclitaxel, which targets spindle mitotic tubes, is the first line of therapy for metastatic breast cancer treatment. Unfortunately, approximately 90% of patients acquire or have an intrinsic drug resistance phenotype in breast cancer (49). Chemoresistance of triple-negative breast cancer against paclitaxel (PAX) is one of the major issues for patients under chemotherapy. Resistance cells to this drug show upregulated genes related to the cell cycle such as E2F3, CDK8, and TLR4. E2F3 and CDK8 directly regulate the cell cycle. TLR4 *via* MAPK and circAMOTL1 *via* AKT in breast cancer cells could heavily affect the cell cycle (50).

#### 4.3.1. CircGFRA1/miR-361-5p/TLR4

High-level expression of Toll-like receptor 4 (TLR4) is associated with poor overall survival of epithelial ovarian cancer patients and metastasis of breast cancer. It has been suggested that TLR4 could activate MyD88 and promote the quiescent state of NF-jB nuclear translocation. High expression of TLR4 in BC lines and tissues diverges with miR-361-5p. MiR-361-5p could target TLR4, and miR-361-5p mimics could positively regulate the viability of paclitaxel-resistant breast cancer cells treated with paclitaxel. By this mechanism, circGFRA1 acts as a sponge to mir-361-5p and controls the sensitivity of cancer cells to paclitaxel. Inhibition of circGFRA1 in an *in vivo* model could inhibit tumor growth with or without paclitaxel (51).

#### 4.3.2. Circ-RNF111/miR-140-5p/E2F3

E2F Transcription Factor 3 (E2F3) is the downstream target of miR-140-5p. In BC, Circ-RNF111 and E2F3 are upregulated in resistance cell lines and cancerous tissues, whereas miR-140-5p is reduced. miR-140-5p directly targets 3UTR of E2F3 and Circ-RNF111 by sponging this microRNA to increase the translation of E2F3 in BC, resulting in increased paclitaxel resistance. Notably, upregulation of mir-140-5p or suppression of E2F3 or Circ-RNF111 could reduce IC_50_, cell viability, colony numbers, cell invasion, and glycolysis of paclitaxel-resistant breast cancer lines (52).

#### 4.3.3. Circ-ABCB10/Let-7a-5p/DUSP7

Circ-ABCB10 is a ceRNA with the ability to sponge Let-7a-5p to regulate DUPS7 (53). Let-7 is one of the most studied microRNAs with significant demoting effects in various cancers by targeting MYC and many other genes in the cell cycle, proliferation, and apoptosis processes (54). It also showed that Let7a could repress various breast cancers, including triple-negative breast cancers *via* HMGA1 and GLUT12. Let-7 could directly bond with DUPS7 mRNA and repress it. Interestingly, accumulation of DUPS7 could inhibit Let7a. In BC lines and tissues, the expression of Circ-ABCB10 and DUSP7 is upregulated, and Let7a is repressed. Furthermore, gain and loss assays showed that Circ-ABCB10 and DUSP7 knockdown could actively increase the susceptibility of BC cells to paclitaxel by activating let-7a, and overexpression of these important molecules could increase PTX sensitivity, apoptosis, and have an inhibitory impact on the invasion and autophagy of PTX-resistant BC cells (53).

#### 4.3.4. CircAMOTL1/AKT

Circular RNA of angiomotin-like 1 (circAMOTL1) is a newly discovered and tempting competing RNA. CircaAMOTL1 could facilitate the expression of AMOTL1 in cervical cancer and promote c-*myc* nuclear translocation in breast cancer (55). This circular RNA is upregulated in tissue and most of the breast cancer cell lines. Its overexpression could increase cell viability, reduction of apoptosis, enhancement of invasion, and paclitaxel resistance. Although the mechanism of action remains under study, overexpression of circAMOTL1 could increase phosphorylated and total AKT protein. Overexpression of this RNA could also increase AKT-related pro-apoptotic (BAX and BAK) and anti-apoptotic (BCL-2) factors, presumably by the accumulation of AKT. circAMOTL1 knockdown, on the other hand, could block this process, which shows a correlation (56).

#### 4.3.5. Circ_0006528/miR-1299/CDK8

As mentioned, Circ_0006528 could induce resistance in BC cells *via* miR-1236-3p/CHD4 (57). This competing endogenous RNA also induces paclitaxel resistance in BC. In paclitaxel-resistant cells, Circ_0006528 by sponging miR-1299 enhances the level of cyclin-dependent kinase 8 (CDK8) proteins. It is noteworthy to know that (CDK8) is upregulated in various cancers, and higher expression of this gene is associated with low surveillance of patients (58). In breast cancer, knockdown of CDK8 impairs EMT and increases tumor cell clearance *via* the CDK8/PDL1 axis. Inhibition of Circ_0006528 restrained proliferation, migration, invasion, and autophagy, whereas it induced apoptosis of PTX-resistant breast cancer cells *in vitro* and impeded the growth of PTX-resistant tumors *in vivo*. This endogenous RNA represses the inhibitory effect of miR-1299 on CDK8. Hence, CDK8 acts as an oncogene. Because CDK8 and Circ_0006528 expression is higher in cancer-resistant cells and miR-1299 expression is lower, the effects of Circ_0006528 could be reversed by mimicry of miR-1299 (57).

### 4.4. Fluorouracil

5-Fluorouracil (5-FU) has been an important anticancer drug to date. With an increase in the knowledge of its mechanism of action, various treatment modalities have been developed over the past few decades to increase its anticancer activity. 5-Fluorouracil metabolism provides 5-FU, which is misincorporated into DNA, generating a mismatch recognized by the MMR system, leading to cell cycle arrest and apoptosis in cases of an irreparable lesion (59). As amazing as this drug is, drug resistance has greatly affected the clinical use of fluorouracil (60).

#### 4.4.1. CDR1as/miR-7/CCNE1

Yang and colleagues discovered the overexpression of CDR1as and its role in 5-FU resistance in BC lines, although its expression in patients remains to be elucidated. They demonstrated that overexpression of CDR1as leads to a reduced amount of miR-7 and overexpression of CCNE1. CCNE1 overexpression has been seen in tumorgenesis and chromosome instability of cells. Furthermore, Yang and colleagues showed that MCF7 and MDA-231-resistant cells transfected with si-CDR1as or miR-7 mimic had decreased IC_50_. These alterations could diminish colony formation rate and increase expression of BAX/BCL and cleaved-caspase-3/caspase-3. These pieces of evidence indicate inhibition of CDR1as and overexpression of miR-7 enhance the chemosensitivity of 5-FU-resistant BC cells. Direct interaction between CDR1as and miR-7 has been shown by an immunoprecipitation assay, and miR7 interaction with the 3-UTR of CCNE1 has been tested *via* a dual luciferase assay. Moreover, this relationship has been shown in the *in vivo* model by knockdown of si-CDR1as and miR-7 (61).

#### 4.4.2. CircFBXL5/miR-216b/HMGA2

High Mobility Group AT-Hook 2 (HMGA2) is a downstream target of miR-216b. Heightened expression of HMGA2 and its various mutations have been seen in countless cancers, especially breast cancer. Of note, overexpression of HMGA2 in breast cancer has a strong association with metastasis (62, 63). The overexpression of CircFBXL5 and reduction of miR-216b have also been seen in breasts, especially MDA-231 and MCF7-resistant cells. Knockdown of CircFBXL5 or introduction of miR-216b mimic could make MDA-231 resistant to 5-FU. Furthermore, the direct interaction of miR-216b with HMGA2 and the inhibitory effects of CircFBXL5 on miR-216 have been seen (64).

## 5. Other drug resistance in breast cancer

Oxaliplatin (OXA) is a third-generation platinum drug used as first-line chemotherapy in colorectal cancer (CRC). Cancer cells acquire resistance to the anticancer drug and develop resistance to this drug by various molecular pathways (65). It has been shown that circFAT1/microRNA-525-5p/SKA1 is involved in the OXA resistance phenotype in breast cancer. CircFAT1 has much higher expression in OXA resistance cancer patients, and its knockdown decreases resistance in breast cancer cell lines. It has been shown that circFAT1 *via* sponging miR-525 could overexpress SKA1, which is a direct target of miR-525. It has been shown that SKA1 can induce Notch and WNT pathways and induce resistance status in cancers (66). Monastrol-resistant breast cancer cells have also been seen. It has been suggested that circRNAMTO1 (hsa circRNA-007874) will be overexpressed in cells treated with monastrol, and this circular RNA could interact with a tumor necrosis factor (TRAF4) and could inhibit the protein level of EG5. However, its role in the resistance cells remains to be elucidated (67). Trastuzumab is a HER2 monoclonal antibody that is used in HER2-positive breast cancer. Although this drug is one of the most effective drugs for the treatment of HER2-positive BC and is part of almost every combined therapy, some overexpressed HER2 patients show mild to high degrees of resistance to this targeted therapy (68). As evidence, the suggested hsa_circ_0001598/miR-1184/PD-L1 pathway could play an important role in this resistance phenotype. Huang and his team showed that circ_0001598 has a higher expression level in BC cancer, especially in trastuzumab-resistant patients. Furthermore, miR-1184 was predicted and validated as a sponging target of hsa_circ_0001598, which results in upregulating PD-L1 as a direct target of hsa_circ_0001598. Although the exact mechanism that drives trastuzumab resistance in cells has not been shown, escaping of CD8 T cells has been seen (69). Regardless of hsa_circ_0001598, exosome-transmitted circHIPK3 from resistant trastuzumab could induce resistance in susceptible cells *via* sponging of various miRNAs (70). Another example of resistance cells to HER2 and, on this occasion, EGFR (HER1) is lapatinib. Lapatinib is a kinase inhibitor that represses phosphorylation of HER1/2 (71). A substantial percentage of patients taking this prescribed drug for advanced stages of breast cancer acquire resistance to lapatinib. It has been shown that circ-MMP11 *via* sponging miR-153 could upregulate ANLN, a critical gene in cell division and metastasis. Interestingly, circ-MMP11 could be transferred *via* exosomes, which might have roles in metastasis and niching (72).

## 6. Discussion

Multidrug resistance is an undesirable feature of cancers. By overcoming this obstacle, the overall quality of treatment could be increased. CircRNA/miRNA/mRNA is a potential cancer treatment target. Circular RNAs are stable RNAs with numerous sponging sites for microRNAs. Since microRNAs could have thousands of targets, the slightest change in this axis could substantially alter the cancer cell network. As most of these RNAs show a restricted correlation to drug resistance to the most important anticancer drugs, they provide countless therapeutic targets to overcome this undesirable feature with the help of RNA interfering technologies such as siRNAs and RNA mimics. In addition, they correlate to the proliferation of cancer cells, even in normal cancer cells versus resistant ones. This phenomenon is observed in almost all of the research reviewed here, and other work might show the significance of these factors in governing cancer cells. These noncoding RNAs could also be used as potential biomarkers to determine the progression and MDR status of cancers, as they have been shown to express differently in resistant cells. Thus, finding and understanding the CircRNA/miRNA/mRNA axis could be a new strategy to identify and overcome drug resistance.

## Author contributions

MG was involved in conceptualization, writing and editing, SB was involved in conceptualization and supervision of project management and supervision. All authors contributed to the article and approved the submitted version.

## Acknowledgments

We thank Dr. Sadegh Safari for his contribution to editing this article.

## Conflict of interest

The authors declare that the research was conducted in the absence of any commercial or financial relationships that could be construed as a potential conflict of interest.

## Publisher’s note

All claims expressed in this article are solely those of the authors and do not necessarily represent those of their affiliated organizations, or those of the publisher, the editors and the reviewers. Any product that may be evaluated in this article, or claim that may be made by its manufacturer, is not guaranteed or endorsed by the publisher.
